# Segmentectomy for patients with early-stage pure-solid non-small cell lung cancer

**DOI:** 10.3389/fonc.2023.1287088

**Published:** 2023-10-31

**Authors:** Atsushi Kamigaichi, Akira Hamada, Yasuhiro Tsutani

**Affiliations:** ^1^ Department of Surgical Oncology, Hiroshima University, Hiroshima, Japan; ^2^ Division of Thoracic Surgery, Department of Surgery, Kindai University, Osaka, Japan

**Keywords:** non-small cell lung cancer, segmentectomy, lobectomy, pure-solid, prognosis, recurrence

## Abstract

For decades, lobectomy has been the recommended surgical procedure for non-small cell lung cancer (NSCLC), including for small-sized lesions. However, two recent pivotal clinical trials conducted by the Japanese Clinical Oncology Group/West Japan Oncology Group (JCOG0802/WJOG4607L) and the Cancer and Leukemia Group B (CALGB140503), which compared the survival outcomes between lobectomy and sublobar resection (the JCOG0802/WJOG4607L included only segmentectomy, not wedge resection), demonstrated the efficacy of sublobar resection in patients with early-stage peripheral lung cancer measuring ≤ 2 cm. The JCOG0802/WJOG4607L demonstrated the superiority of segmentectomy over lobectomy with respect to overall survival, implying the survival benefit conferred by preservation of the lung parenchyma. Subsequently, the JCOG1211 also demonstrated the efficacy of segmentectomy, even for NSCLC, measuring up to 3 cm with the predominant ground-glass opacity phenotype. Segmentectomy has become the standard of care for early-stage NSCLC and its indications are expected to be further expanded to include solid lung cancers > 2 cm. However, local control is still a major concern for segmentectomy for higher-grade malignant tumors. Thus, the indications of segmentectomy, especially for patients with radiologically pure-solid NSCLC, remain controversial due to the aggressive nature of the malignancy. In this study, we reviewed previous studies and discussed the efficacy of segmentectomy for patients with such tumors.

## 1 Introduction

In 1995, a randomized prospective trial conducted by the Lung Cancer Study Group (LCSG) reported that sublobar resection resulted in poorer survival rates with a higher recurrence rate compared to lobectomy in patients with early-stage non-small cell lung cancer (NSCLC) (1). Subsequently, lobectomy has been established as the standard surgical procedure for NSCLC, even for cases involving small-sized lesions. However, recent developments in clinical staging modalities, such as thin-section computed tomography (CT) and 18-fluoro-2-deoxyglucose positron emission tomography/computed tomography, have enhanced the detection of small-sized early-stage lung cancers and the diagnostic accuracy of clinical staging of NSCLC. Concurrently, some recent pivotal clinical trials conducted by the Japanese Clinical Oncology Group (JCOG), West Japan Oncology Group (WJOG), and the Cancer and Leukemia Group B (CALGB) have demonstrated the efficacy of sublobar resection compared to lobectomy in patients with early-stage small-sized NSCLC (2, 3). The JCOG0802/WJOG4607L trial demonstrated the superiority of segmentectomy over lobectomy in terms of overall survival (OS) and similar recurrence-free survival (RFS) in patients with radiologically solid-predominant peripheral small-sized NSCLC measuring ≤ 2 cm (2). Segmentectomy has garnered considerable attention due to its reduced toxicity and the improved survival benefits associated with lung parenchyma preservation.

Radiologically pure-solid NSCLC, lacking ground-glass opacity (GGO) components, represents a highly malignant neoplasm with worse prognoses compared to part-solid NSCLC containing GGO components (4–10). Consequently, concerns persist regarding certain disadvantages of segmentectomy, including the risk of postoperative recurrence. Therefore, the indication of segmentectomy, especially for patients with radiologically pure-solid NSCLC, remains controversial, necessitating further discussion on the appropriate treatment strategy for radiologically pure-solid tumors.

This study reflected on the evolving attitudes toward segmentectomy, reviewing previous studies, and evaluating the efficacy of segmentectomy for patients with early-stage radiologically pure-solid NSCLC. Moreover, we discussed the possibility of further expansion of the surgical indications of segmentectomy in the context of the new era of lung cancer surgery after the JCOG/WJOG and CALGB trials.

## 2 Transition in views on sublobar resection

Until the publication of the JCOG0802/WJOG4607L study, the only confirmatory phase III trial comparing lobectomy and sublobar resection was that conducted by the LCSG in North America (1). This trial enrolled 276 patients with stage IA NSCLC measuring ≤ 3 cm between February 1982 and November 1988. The results showed a 5-year survival rate of 63% in the lobectomy group versus 42% in the sublobar resection group (*P* = 0.088), indicating that sublobar resection is inferior to lobectomy. In addition, the rate of local recurrence was lower in the lobectomy group (6%) compared to the sublobar resection group (18%) (*P* = 0.008). Thus, based on the inferences from this trial, lobectomy served as a standard surgical procedure for patients with clinical stage IA NSCLC, and this practice has been followed until today.

However, the LCSG trial had some limitations. First, the accuracy of clinical staging was low due to the poor quality of imaging (posteroanterior and lateral chest radiography were mainly used). Second, clinical-stage IA NSCLC was considered to have a potential risk of unsuspected lymph node metastasis. Nevertheless, the sublobar resection arm included not only segmentectomy but also wedge resection without lymph node dissection. Third, non-peripheral tumors were also considered to be included; thus, sublobar resection for such tumors may not have ensured adequate surgical margins.

Because of these limitations, it was questionable whether lobectomy should continue to be the standard surgical procedure for early-stage NSCLC.

**Figure 1:**
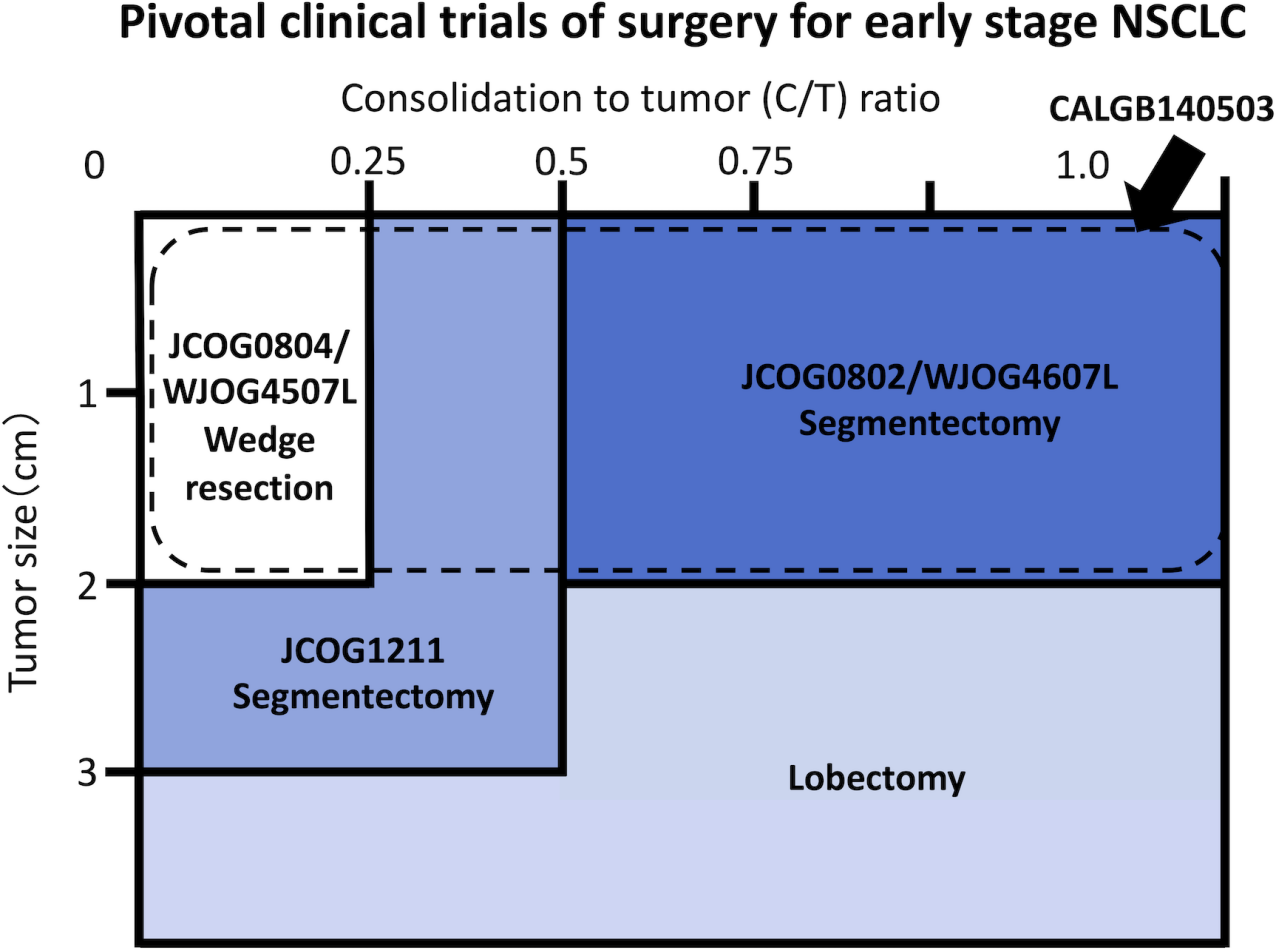
Schema of pivotal clinical trials conducted by the Japan Clinical Oncology Group (JCOG), West Japan Oncology Group (WJOG), and Cancer and Leukemia Group B (CALGB) Study.

The JCOG0201 investigated the association between radiological findings and prognosis in patients with early-stage NSCLC to define radiologically non-invasive NSCLC (11). It defined radiologically non-invasive lung cancer as the presence of a maximum tumor diameter of 2 cm with a consolidation-to-tumor (C/T) ratio of ≤ 0.25, which was consequently changed to ≤ 0.5 due to its excellent prognosis (11). Based on the results of the JCOG0201 and specific features of sublobar resection, three confirmatory clinical trials investigating the efficacy of sublobar resection have been conducted in Japan: i.e., the JCOG0804/WJOG4507L (12), JCOG1211 (13), and JCOG0802/WJOG4607L (2) (**Figure 1**).

The JCOG0804/WJOG4507L was a single-arm confirmatory trial conducted to evaluate the efficacy and safety of sublobar resection for GGO-predominant peripheral NSCLC sized ≤ 2.0 cm with a C/T ratio ≤ 0.25 (12, 14). The JCOG1211 aimed to evaluate the efficacy and safety of segmentectomy for GGO-predominant NSCLC up to 3 cm in size (13). The JCOG0802/WJOG4607L was a randomized controlled non-inferiority trial comparing segmentectomy and lobectomy for radiologically solid predominant NSCLC sized ≤ 2 cm. In addition, the CALGB140503 was conducted in North America to compare lobectomy and sublobar resection, including segmentectomy and wedge resection, for NSCLC sized ≤ 2 cm, excluding pure ground-glass nodule (GGN) (**Figure 1**) (3).

In summary, all four trials demonstrated the efficacy of sublobar resection for small-sized NSCLC. Currently, preserving the lung parenchyma has become a global surgical trend for patients with early-stage NSCLC.

## 3 Tumor malignancy and prognosis of radiologically pure-solid and part-solid NSCLC

To date, thin-section CT is the optimal diagnostic modality for evaluating tumor malignancy and the invasiveness of early-stage NSCLC (11). The GGO component is a radiologically non-invasive area (11). Based on the presence of the GGO component on thin-slice CT, lung tumors are classified into radiologically pure-solid NSCLC without the GGO component, part-solid NSCLC with GGO, and pure GGN. Pure-solid NSCLC shows higher pathological invasiveness, including lymphatic invasion, vascular invasion, lymph node metastasis, spread through air spaces (STAS), and lymph node involvement compared to part-solid NSCLC with a GGO component (4–10). The supplementary analysis of JCOG0201 also showed worse OS in patients with radiologically pure-solid NSCLC compared to those with part-solid NSCLC (5). Thus, radiologically pure-solid NSCLC is oncologically highly invasive and has a worse prognosis than part-solid NSCLC. Even the presence of a small GGO component is also reported to be associated with a favorable prognosis in patients with NSCLC measuring ≤ 2 cm (8, 9). Although the favorable impact of a small GGO component on malignant potential in NSCLC measuring > 2–3 cm is controversial, there is no doubt that radiologically pure-solid NSCLC has highly malignant characteristics (8, 9).

Furthermore, the validity of segmentectomy for patients with unsuspected lymph node metastasis is debatable (15, 16); however, unsuspected lymph node metastasis is a major concern since residual tumors could affect the outcome of sublobar resection. The frequency of unsuspected lymph node metastasis is reportedly 11.1–17.7% for clinical stage IA1-2 pure-solid NSCLC and 17.3–36.0% for clinical stage IA3 pure-solid NSCLC (8, 10, 17). On the other hand, the risk of lymph node metastasis reportedly depends on tumor location, i.e., central or peripheral, rather than the malignancy of the tumor itself (18, 19). The frequency of unsuspected lymph node metastasis was lower in peripherally located radiologically pure-solid NSCLC (≤ 2 cm: 7.8% and > 2–3 cm: 13.3%), which are generally candidates for sublobar resection, compared to their centrally located counterparts (≤ 2 cm: 29.8% and > 2–3 cm: 20.3%) (19). Regarding unsuspected hilar lymph node metastasis, the frequency in peripherally located tumors (≤ 2 cm: 6.7% and > 2–3 cm: 8.3%) was also lower than that in centrally located tumors (≤ 2 cm: 24.6% and > 2–3 cm: 17.4%) (19). Moreover, the frequency of hilar lymph node metastasis did not differ significantly between radiologically pure-solid NSCLC located in the peripheral lung fields measuring > 2–3 cm (8.3%) and those measuring ≤ 2 cm (6.7%) (19).

## 4 Prognostic impact of segmentectomy on patients with pure-solid NSCLC measuring ≤ 2 cm

### 4.1 Previous retrospective studies reporting the efficacy of segmentectomy for patients with pure-solid NSCLC measuring ≤ 2 cm

After the LCSG trial, several retrospective studies investigated the efficacy of segmentectomy for small-sized NSCLC ≤ 2 cm. Although there were concerns about the worsening of survival with the increase in local recurrence due to the highly malignant characteristics of radiologically pure-solid NSCLC, some of these retrospective studies reported the efficacy of segmentectomy for this type of NSCLC (**Table 1**) (20–24). Most studies indicated comparable survival outcomes, including OS and RFS, between segmentectomy and lobectomy for patients with radiologically pure-solid NSCLC sized ≤ 2 cm (20, 22–24), although one study reported worse locoregional recurrence-free survival in the segmentectomy arm (3-year rate, 82.2%) compared to the lobectomy arm (3-year rate, 90.6%, *P* = 0.0488) (21).

**Table 1:** Summary of previous studies comparing segmentectomy and lobectomy for early-stage radiologically pure-solid NSCLC measuring ≤ 2 cm.

Author	Year	Study design	Seg (n)	Lob (n)	5-year OS (Seg vs. Lob)	5-year RFS (Seg vs. Lob)	Recurrence pattern
Koike et al. (20)	2016	Retrospective	87	87	84.0% vs. 85.0%, *P* = 0.767	77.0% vs. 80.0%, *P* = 0.635	Locoregional only: Seg: 6.9% Lob: 5.7% Distant only: Seg: 12.6% Lob: 13.8% Both: Seg: 3.4% Lob: 0%
Hattori et al. (21)	2017	Retrospective	29	183	3-year OS 93.1 vs 91.1%, *P* = 0.9491	3-year Locoregional RFS 82.2 vs 90.6%, *P* = 0.0488	N/A
Tsubokawa et al. (22)	2018	Retrospective	52	44	94.2% vs. 92.0% *P* = 0.723	84.1% vs. 82.2% *P* = 0.745 HR: 1.11 (0.40–3.06)	Locoregional only: Seg: 1.9% Lob: 9.1% Distant only: Seg: 5.8% Lob: 6.8% Both: Seg: 3.8% Lob: 0%
Soh et al. (23)	2022	Retrospective	346	1505	80.3% vs. 84.2% *P* = 0.080	74.0% vs. 75.0% *P* = 0.73	N/A
Zhihua et al. (24)	2023	Retrospective	98	246	97.8% vs. 90.0% *P* = 0.028 HR: 0.36 (0.08–1.59)	92.4% vs. 81.1% *P* = 0.011 HR: 0.72 (0.30–1.77)	Locoregional: Seg: 4.08% Lob: 2.85% Distant: Seg: 1.02% Lob: 10.57%
Saji et al. (2) Hattori et al. (25)	2022	RCT (Subgroup analysis)	279	274	92.4% vs. 86.1%, *P* = 0.0333 HR: 0.641 (0.424–0.969)	82.0% vs. 81.7% *P* = 0.9420 HR: 1.013 (0.723–1.417)	Locoregional only: Seg: 10.7% Lob: 4.4% Distant only: Seg: 1.8% Lob: 4.7% Both: Seg: 5.0% Lob: 3.3%

HR, hazard ratio; Lob, lobectomy; NSCLC, non-small cell lung cancer; N/A, not available; OS, overall survival; RCT, randomized controlled trial; RFS, recurrence-free survival; Seg, segmentectomy.

### 4.2 The efficacy of segmentectomy for patients with pure-solid NSCLC in the JCOG0802/WJOG4607L

The only confirmatory trial comparing segmentectomy to lobectomy in patients with small-sized NSCLC was the JCOG0802/WJOG4607L (2). This trial demonstrated not only the non-inferiority but also the superiority of segmentectomy over lobectomy with respect to OS in patients with peripherally located early-stage solid predominant NSCLC ≤ 2 cm. Although the rate of mortality due to primary disease was comparable between the segmentectomy and lobectomy groups, the rate of mortality due to other diseases, including second cancer, was lower in the segmentectomy group than in the lobectomy group. A greater proportion of patients in the segmentectomy group underwent curative surgery for a second primary cancer or postoperative local recurrence compared to that in the lobectomy group. These results imply that merely improving local control does not improve survival in patients with early-stage NSCLC, and preserving the lung parenchyma may have prolonged survival after lung surgery. In addition, in the JCOG0802/WJOG4607L, the finding of thin-section CT (solid/part solid) was set as a stratification factor, which formed the basis of the subgroup analysis. A greater survival benefit of segmentectomy was observed in the radiologically pure-solid NSCLC group compared to the part-solid NSCLC group [pure-solid group: hazard ratio (HR): 0.641 (95% confidence interval 0.424–0.969) and part-solid group: HR: 0.733 (95% confidence interval 0.413–1.301)] (2). A history of smoking was more frequent in patients with radiologically pure-solid NSCLC compared to those with part-solid NSCLC (10, 17, 26, 27). The proportion of patients with decreased lung function and those who developed a second disease, such as second primary cancer, was expected to be higher in patients with pure-solid NSCLC (28–30). Therefore, the survival benefit of preserving the lung parenchyma by segmentectomy was considered to manifest more in patients with radiologically pure-solid NSCLC. Furthermore, detailed data on the supplemental analysis of the JCOG0802/WJOG4607L, which investigated the survival of segmentectomy compared to lobectomy for radiologically pure-solid NSCLC, was presented at the 103^rd^ Annual Meeting of the American Association for Thoracic Surgery (25). In the supplemental analysis, local recurrence occurred more frequently in the segmentectomy group (16.1%) than in the lobectomy group (7.7%). However, the RFS of segmentectomy was comparable to that of lobectomy [HR: 1.013 (95% confidence interval 0.723–1.417)]. In addition, the rate of mortality due to diseases other than primary lung cancer was higher in the lobectomy group (12.0%) than in the segmentectomy group (5.7%). Although this was a subgroup analysis, segmentectomy may provide survival benefits for patients with oncologically higher-grade tumors. Indeed, previous studies have demonstrated the efficacy of segmentectomy even for more aggressive hypermetabolic tumors or pathologically invasive cancers (31, 32).

The accuracy of lymph node dissection in sublobar resection is often debated, especially for lung cancers with a potentially high risk of unsuspected lymph node metastasis. In contrast, the JCOG0802/WJOG4607L trial found no difference in the frequency of nodal upstaging or hilar lymph node recurrence between the segmentectomy and lobectomy groups (2). This indicates that lymph node dissection could be performed adequately even with segmentectomy, although there are hilar lymph nodes that are difficult to dissect with segmentectomy. Furthermore, according to the JCOG0802/WJOG4607L, locoregional recurrence in the mediastinal lymph nodes was more frequent with segmentectomy compared to lobectomy (2). Even with segmentectomy, sufficient mediastinal lymph node dissection should be performed to avoid residual tumor cells and achieve accurate nodal staging (33). The results of the JCOG1413, which investigated the clinical efficacy of lobe-specific nodal dissection for clinical stage I–II NSCLC, may provide insights on the extent of mediastinal lymph node dissection (i.e., lobe-specific or systematic nodal dissection) (34).

### 4.3 The efficacy of sublobar resection for small-sized NSCLC reported in the CALGB140503

Furthermore, the CALGB140503 reported the non-inferiority of sublobar resection, including segmentectomy (40.9%) and wedge resection (59.1%), compared to lobectomy for patients with early-stage NSCLC measuring ≤ 2 cm with respect to disease-free survival (DFS) (3). Despite the lack of information on the CT findings (solid/part solid), the population of the CALGB140503 showed a worse prognosis (5-year DFS: 63.6% and 5-year OS: 80.3% in the sublobar resection group, 5-year DFS: 64.1% and 5-year OS: 78.9% in the lobectomy group) compared to that of the JCOG0802/WJOG4607L. In addition, an unplanned *post hoc* analysis, albeit statistically underpowered, showed that survival did not differ between segmentectomy and lobectomy (35).

Thus, we can infer that segmentectomy should be considered the standard procedure even for radiologically pure-solid lung cancer, although care must be taken to prevent local recurrence.

## 5 Expansion of the indications for segmentectomy to patients with radiologically pure-solid NSCLC > 2–3 cm

As mentioned above, segmentectomy has become a standard surgical procedure even for early-stage pure-solid NSCLC measuring ≤ 2 cm, and its indications are expected to be further expanded to include solid lung cancers measuring > 2 cm. However, radiologically pure-solid NSCLC measuring > 2–3 cm was not included in these trials. Thus, the only confirmatory trial that included patients with radiologically pure-solid NSCLC sized > 2–3 cm in the study population was the LCSG trial (1). Therefore, lobectomy remains the standard procedure for patients with these tumors.

Recently, the JCOG1211 demonstrated the efficacy of segmentectomy even for NSCLC measuring up to 3 cm with GGO predominance (13). This trial indicated that segmentectomy is technically feasible for tumors measuring > 2–3 cm. Thus, based on the aggregate technical feasibility and survival benefit of segmentectomy proven in the prospective trials, the need to clarify the oncological suitability of segmentectomy for radiologically pure-solid tumors sized > 2–3 cm has gained traction.

### 5.1 Retrospective studies reporting the efficacy of segmentectomy for patients with pure-solid NSCLC measuring > 2–3 cm

**Table 2** shows the summary of previous studies comparing segmentectomy and lobectomy for patients with early-stage NSCLC measuring > 2–3 cm that were considered to include radiologically pure-solid tumors in the study population. The indications of segmentectomy include curative intent for patients who are fit to undergo lobectomy and passive intent for compromised patients who are unfit to undergo lobectomy. Basically, segmentectomy is performed with passive intent for patients with radiologically solid NSCLC measuring > 2–3 cm. Therefore, the survival results should be interpreted with caution due to the potential inconsistency of the patients’ backgrounds. Nevertheless, some studies have reported the feasibility of segmentectomy for patients with such tumors (23, 36, 37, 39, 41, 42, 44).

**Table 2:** Summary of previous studies comparing segmentectomy and lobectomy for patients with early-stage NSCLC sized > 2–3 cm that were considered to include radiologically pure-solid NSCLC in the study population.

Authors	Year	Seg (n)	Lob (n)	C/T ratio	Survival (Seg vs. Lob)	Recurrence pattern
Okada et al. (36)^p^	2005	76	268	N/A	5-year CSS: 84.6% vs 87.4%	N/A
Carr et al. (37)^p^	2012	57	88	N/A	RFS: *P* = 0.423	Locoregional Seg: 5.3% Lob: 9.0% Distant Seg: 10.5% Lob: 20.5%
Deng et al. (38)^p^	2014	31	93	N/A	5-year OS: 55.8% vs. 77.6%, *P* = 0.05 5-year RFS: 54.1% vs. 74.7%, *P* = 0.05	N/A
Landreneau et al. (39)^c^	2014	N/A	N/A	N/A	TTR: *P* = 0.395	N/A
Cao et al. (40)^p^	2018	221 (PSM: 221)	5257 (PSM: 221)	N/A	Whole cohort OS: HR 1.698 (95% CI, 1.395–2.066), *P* < 0.001 PSM OS: HR 1.63 (95% CI, 1.210–2.197), *P* = 0.001	N/A
Kamigaichi et al. (41)^c^	2020	43 (PSM: 37)	154 (PSM: 37)	1 [IQR, 0.8–1.0]	Whole cohort 5-year OS: 90.6% vs. 80.0%, *P* = 0.42 5-year RFS: 82.7% vs. 73.4%, *P* = 0.30 PSM 5-year OS: 89.3% vs. 82.9% 5-year RFS: 80.1% vs. 79.5%	N/A
Chan et al. (42)^c^	2021	90 (PSM: 90)	276 (PSM: 90)	N/A	Whole cohort OS: HR 1.07 (95% CI, 0.74–1.52), *P* = 0.73 RFS: HR 1.19 (95% CI, 0.58–1.66), *P* = 0.32 TTR: HR 1.24 (95% CI, 0.78–1.97), *P* = 0.37 PSM OS: HR 1.23 (95% CI, 0.91–1.82), *P* = 0.17 RFS: HR 1.23 (95% CI, 0.82–1.85), *P* = 0.32 TTR: HR 0.95 (95% CI, 0.52–1.73), *P* = 0.87	N/A
Peng et al. (43)^p^	2022	945 (CDCI of 0 : 411)	18990 (CDCI of 0 : 9420)	N/A	Whole cohort 5-year OS 54.3% vs. 64.9%, *P* < 0.0001 CDCI of 0 5-year OS 64.3% vs. 69.6%, *P* = 0.133	N/A
Soh et al. (23)^c^	2022	102	1460	1	5-year OS: 70.0% vs. 79.2%, *P* = 0.077 5-year RFS: 63.1% vs. 67.1%, *P* = 0.39	N/A
Kamigaichi et al. (44)^c^	2023	44 (PSM: 41)	368 (PSM: 41)	1	CIR: HR 1.04 (95% CI, 0.48–2.30), *P* = 0.91 Whole cohort 5-year CIR: 21.9% vs. 20.8%, *P* = 0.88 PSM 5-year CIR: 20.6% vs. 22.8%, *P* = 0.55	Locoregional Seg: 6.8%, Lob: 9.0% Distant Seg: 11.4% Lob: 15.2%

*All studies were retrospective in design.

c The study population were defined by clinical staging.

p The study population were defined by pathological staging.

CDCI, Charlson-Deyo Comorbidity Index Score; CIR, cumulative incidence of recurrence; CSS, cancer-specific survival; C/T ratio, consolidation-to-tumor ratio; HR, hazard ratio; Lob, lobectomy; N/A, not available; NSCLC, non-small cell lung cancer; OS, overall survival; PSM, propensity-score matching; RCT, randomized control trial; RFS, recurrence-free survival; Seg, segmentectomy; TTR, time to recurrence.

Only two of these retrospective studies provided the results of the comparison of segmentectomy and lobectomy for radiologically pure-solid NSCLC (23, 44). A large-scale study using the Japanese Joint Committee of Lung Cancer Registry Database reported that segmentectomy tended to yield worse OS (*P* = 0.077) and DFS (*P* = 0.39) than lobectomy in patients with radiologically pure-solid clinical stage IA3 NSCLC, although the difference was not statistically significant (23). However, multivariable analysis adjusted for factors of patient background, such as performance status, comorbidities, and respiratory function, revealed that segmentectomy yielded survival outcomes (OS: HR, 1.177; 95% CI, 0.082–1.727; *P* = 0.405; DFS: HR, 1.055; 95% CI, 0.750–1.484; *P* = 0.758) comparable to those of other surgical procedures, including mainly lobectomy (lobectomy, 93.1%; wedge resection, 6.9%). Moreover, retrospective studies conducted by Kanagawa Cancer Center, Tokyo Medical University, and Hiroshima University found no significant difference in the recurrence risk and recurrence patterns between segmentectomy and lobectomy in patients with radiologically pure-solid NSCLC measuring > 2–3 cm (44). Although there is no information on the CT findings (solid/part solid), a single-center prospective study conducted at Kumamoto University, which included 31 patients with clinical T1cN0M0 NSCLC, reported the long-term prognosis after segmentectomy for clinical T1N0M0 NSCLC (45). The 10-year OS, recurrence-free probability, and local recurrence-free probability rates after segmentectomy in patients with clinical T1cN0M0 NSCLC were 75%, 69%, and 85%, respectively. Moreover, 3 of 31 patients (9.7%) with clinical T1cN0M0 NSCLC developed local recurrence (surgical margin recurrence in 2 patients and preserved lobe recurrence in 1 patient) after segmentectomy. However, these patients underwent additional treatment, such as lobectomy or radiation, for local recurrence. Consequently, no patient succumbed to primary NSCLC (45).

A study indicated that segmentectomy was inferior to lobectomy in patients with NSCLC measuring > 2–3 cm, but the prognosis of segmentectomy was comparable to lobectomy only in a subpopulation with a Charlson-Deyo Comorbidity Index score of 0 (43). On the other hand, only studies that did not adjust for patients’ backgrounds suggested that segmentectomy was unsuitable for patients with NSCLC > 2–3 cm (38, 40). However, selection bias must be carefully considered while interpreting the results of these studies. Cao et al. attempted to minimize potential bias by employing propensity score-matched analysis, but it was insufficient to match the tumor and patient backgrounds between the lobectomy and segmentectomy groups (40).

### 5.2 Local control for patients with pure-solid NSCLC measuring > 2–3 cm

While segmentectomy may yield comparable survival to lobectomy even in patients with radiologically pure-solid NSCLC > 2–3 cm, local control is a major concern for larger and higher-grade tumors. Ensuring sufficient surgical margins and adequate lymph node dissection are crucial to preventing locoregional recurrence. The presence of STAS and a micropapillary component, which could be risk factors for margin recurrence after segmentectomy, should be considered (46). STAS was observed in 22% of patients with radiologically pure-solid NSCLC measuring > 2–3 cm (17). However, in previous studies, the prognosis was similar between segmentectomy and lobectomy for NSCLC with STAS if the surgical margin was adequate. According to these studies, a surgical margin ≥ 20 mm could prevent postoperative recurrence in the presence of STAS (47, 48). Furthermore, one study reported that 36% of lung adenocarcinomas > 2–3 cm included histopathologically micropapillary or solid subtypes (49). According to previous studies, surgical margins ≥ 10 mm could contribute to the decreased risk of local recurrence in lung adenocarcinomas, including these histological subtypes (50). By securing a sufficient surgical margin with appropriate lymph node dissection, segmentectomy may be suitable for larger, high-grade tumors, namely radiologically pure-solid NSCLC sized > 2–3 cm.

Sublobar resection includes not only segmentectomy but also wedge resection. Previous studies have indicated that cancer control was better in patients who underwent segmentectomy than those who underwent wedge resection for clinical stage IA NSCLC (51–53). Segmentectomy is an anatomic resection that can dissect the hilar lymph nodes, while wedge resection is a nonanatomic procedure that cannot dissect the hilar lymph nodes. Thus, lymph nodes are not as adequately evaluated by wedge resection as segmentectomy. Furthermore, although wedge resection was adopted for NSCLC sized ≤ 2 cm in the CALGB140503, it may be difficult to secure a sufficient surgical margin by wedge resection for tumors > 2 cm. Thus, wedge resection may be unsuitable for radiologically pure-solid NSCLC sized > 2–3 cm. A randomized phase III trial (JCOG1909) is currently underway to confirm the superiority of segmentectomy over wedge resection for patients with clinical Stage IA NSCLC with poor pulmonary reserve or other major comorbidities that are contraindications for lobectomy but can tolerate sublobar resection (high-risk operable) (54). The results of this trial will also provide insights into the difference in cancer control between segmentectomy and wedge resection for NSCLC measuring > 2–3 cm.

## 6 Discussion

As a result of the recent JCOG/WJOG and CALGB trials, the validity of sublobar resection became widely recognized, making sublobar resection for early-stage small-sized NSCLC a mainstream procedure worldwide. Although it was thought that segmentectomy may not be suitable for radiologically pure-solid NSCLC, several studies, such as the subgroup analysis of the JCOG0802/WJOG4607L, demonstrated the efficacy of segmentectomy even for radiologically pure-solid NSCLC (2, 20, 22–25). Based on these results, segmentectomy is expected to become the standard surgical procedure even for patients with radiologically pure-solid NSCLC sized ≤ 2 cm. As its less invasive nature, segmentectomy was reported to contribute to the preservation of postoperative respiratory function (2, 41, 55), nutritional status (56), and a reduction of the risk of postoperative complications compared to lobectomy (57). Above all, the fact that the frequency of other causes of death was lower in the segmentectomy group in the JCOG0802/WJOG4607L is a robust argument supporting the less invasive nature of segmentectomy (2). Clinicians should provide patients with lung surgeries that minimize invasion of the patient’s physical function while achieving curative treatment of the cancer.

Despite the survival benefits associated with the preservation of the lung parenchyma, local control is a major concern in the expansion of the indications of segmentectomy for larger, high-grade tumors. Appropriate evaluation of sufficient surgical margins and lymph node status is crucial to preventing local recurrence after segmentectomy. If these objectives can be achieved, segmentectomy may become a suitable treatment modality for radiologically pure-solid NSCLC sized > 2–3 cm. However, future confirmatory clinical trials are warranted.

## Author contributions

AK: Conceptualization, Data curation, Investigation, Methodology, Writing – original draft. AH: Conceptualization, Data curation, Investigation, Supervision, Writing – review & editing. YT: Conceptualization, Data curation, Investigation, Methodology, Supervision, Writing – review & editing.
